# Quantum Models for Psychological Measurements: An Unsolved Problem

**DOI:** 10.1371/journal.pone.0110909

**Published:** 2014-10-24

**Authors:** Andrei Khrennikov, Irina Basieva, Ehtibar N. Dzhafarov, Jerome R. Busemeyer

**Affiliations:** 1 Department of Mathematics, Linnaeus University, Växjö, Sweden; 2 Department of Psychological Sciences, Purdue University, West Lafayette, Indiana, United States of America; 3 Department of Psychological and Brain Sciences, Indiana University, Bloomington, Indiana, United States of America; The Ohio State University, Center for Cognitive and Brain Sciences, Center for Cognitive and Behavioral Brain Imaging, United States of America

## Abstract

There has been a strong recent interest in applying quantum theory (QT) outside physics, including in cognitive science. We analyze the applicability of QT to two basic properties in opinion polling. The first property (response replicability) is that, for a large class of questions, a response to a given question is expected to be repeated if the question is posed again, irrespective of whether another question is asked and answered in between. The second property (question order effect) is that the response probabilities frequently depend on the order in which the questions are asked. Whenever these two properties occur together, it poses a problem for QT. The conventional QT with Hermitian operators can handle response replicability, but only in the way incompatible with the question order effect. In the generalization of QT known as theory of positive-operator-valued measures (POVMs), in order to account for response replicability, the POVMs involved must be conventional operators. Although these problems are not unique to QT and also challenge conventional cognitive theories, they stand out as important unresolved problems for the application of QT to cognition. Either some new principles are needed to determine the bounds of applicability of QT to cognition, or quantum formalisms more general than POVMs are needed.

## Introduction

Quantum theory (QT) is the mathematical formalism of quantum physics. (Sometimes the two are considered synonymous, in which case what we call here QT would have to be called “mathematical formalism of QT.”) However, QT has recently begun to be used in various domains outside of physics, in biology, economics, and cognitive science (see Text S1 Representative Bibliography). For overviews, see the recently published monographs [1] and [2], as well as the recent target article in *Brain and Behavioral Sciences* [3] with ensuing commentaries and rejoinders. There is one obvious similarity between cognitive science and quantum physics: both deal with observations that are fundamentally probabilistic. This similarity makes the use of QT in cognitive science plausible, as QT is specifically designed to deal with random variables. Here, we analyze the applicability of QT in opinion-polling, and compare it to psychophysical judgments.

On a very general level, QT accounts for the probability distributions of measurement results using two kinds of entities, called *observables* 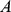 and *states* 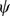 (of the system on which the measurements are made). Let us assume that measurements are performed in a series of consecutive trials numbered 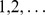. In each trial 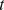 the experimenter decides what measurement to make (e.g., what question to ask), and this amounts to choosing an observable 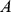. Despite its name, the latter is not observable per se, in the colloquial sense of the word, but it is associated with a certain set of values 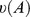, which are the possible results one can observe by measuring 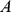. In a psychological experiment these are the responses that a participant is allowed to give, such as *Yes* and *No*.

The probabilities of these outcomes in trial 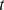 (conditioned on all the previous measurements and their outcomes) are computed as some function of the observable 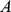 and of the state 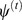 in which the system (a particle in quantum physics, or a participant in psychology) is at the beginning of trial 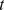, 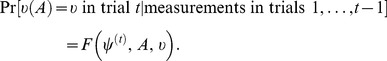
(1)


This measurement changes the state of the system, so that at the end of trial 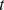 the state is 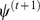, generally different from 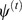. The change 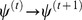 depends on the observable 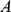, the state 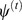, and the value 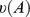 observed in trial 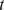, 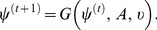
(2)


On this level of generality, a psychologist will easily recognize in (1)–(2) a probabilistic version of the time-honored Stimulus-Organism-Response (S-O-R) scheme for explaining behavior [4]. This scheme involves stimuli (corresponding to 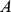), responses (corresponding to 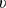), and internal states (corresponding to 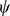). It does not matter whether one simply identifies 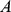 with a stimulus, or interprets 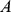 as a kind of internal representation thereof, while interpreting the stimulus itself as part of the measurement procedure (together with the instructions and experimental set-up, that are usually fixed for the entire sequence of trials). What is important is that the stimulus determines the observable 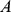 uniquely, so that if the same stimulus is presented in two different trials 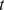 and 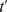, one can assume that 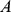 is the same in both of them.

The state 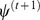 determined by (2) may remain unchanged between the response 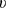 terminating trial 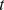 and the presentation of (the stimulus corresponding to) the new observable that initiates trial 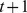. In some applications this interval can indeed be negligibly small or even zero, but if it is not, one has to allow for the evolution of 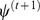 within it. In QT, the “pure” evolution of the state (assuming no intervening inter-trial inputs) is described by some function 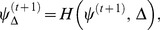
(3)where 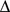 is the time interval between the recording of 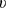 in trial 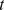 and the observable in trial 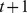. This scheme is somewhat simplistic: one could allow 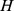 to depend, in addition to the time interval 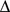, on the observable 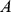 and the outcome 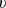 in trial 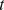. We do not consider such complex inter-trial dynamics schemes in this paper.

The reason we single out opinion-polling and compare it to psychophyscis is that they exemplify two very different types of stimulus-response relations.

In a typical opinion-polling experiment, a group of participants is asked one question at a time, e.g., *a* =  “Is Bill Clinton honest and trustworthy?” and *b* =  “Is Al Gore honest and trustworthy?” [5]. The two questions, obviously, differ from each other in many respects, none of which has anything to do with their content: the words “Clinton” and “Gore” sound different, and the participants know many aspects in which Clinton and Gore differ, besides their honesty or dishonesty. Therefore, if a question, say, 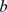, were presented to a participant more than once, she would normally recognize that it had already been asked, which in turn would compel her to repeat it, unless she wants to contradict herself. One can think of situations when the respondent can change her opinion, e.g., if another question posed between two replications of the question provides new information or reminds something forgotten. Thus, if the answer to the question *a* =  “Do you want to eat this chocolate bar?” is Yes, and the second question is *b* =  “Do you want to lose weight?,” the replications of 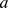 may very well elicit response No. It is even conceivable that if one simply repeats the chocolate question twice, the person will change her mind, as she may think the replication of the question is intended to make her “think again.” In a wide class of situations, however, changing one’s response would be highly unexpected and even bizarre (e.g., replace 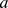 in the example above with “Do you like chocolate?”). We assume that the pairs of questions asked, e.g., in Moore’s study [5] are of this type.

In a typical psychophysical task, the stimuli used are identical in all respects except for the property that a participant is asked to judge. Consider a simple detection paradigm in which the observer is presented one stimulus at a time, the stimulus being either 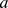 (containing a signal to be detected) or 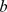 (the “empty” stimulus, in which the signal is absent). For instance, 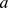 may be a tilted line segment, and 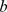 the same line segment but vertical, the tilt (which is the signal to be detected) being too small for all answers to be correct. Clearly, the participant in such an experiment cannot first decide that the stimulus being presented now has already been presented before, and that it has to be judged to be 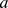 because so it was before.

With this distinction in mind, however, the formalism (1)–(2)–(3) can be equally applied to both types of situations. In both cases 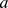 is to be replaced with some observable 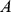, and 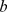 with some observable 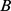 (after which 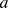 and 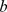 per se can be forgotten). The values of 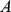 and 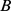 are the possible responses one records. In the psychophysical example, 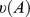 and 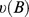 each can attain one of two values: 1 =  “I think the stimulus was tilted” or 0 =  “I think the stimulus was vertical”. The psychophysical analysis consists in identifying the hit-rate and false-alarm-rate functions (conditioned on the previous stimuli and responses) 
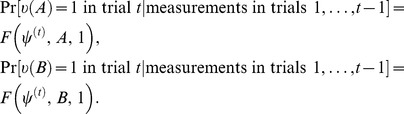
(4)


The learning (or sequential-effect) aspect of such analysis consists in identifying the function 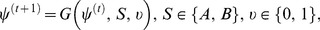
(5)combined with the “pure” inter-trial dynamics (3).

In the opinion-polling example (say, about Clinton’s and Gore’s honesty), there are two hypothetical observables: 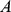, corresponding to the question *a* =  “Is Bill Clinton honest?”, and 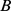, corresponding to the question *b* =  “Is Al Gore honest?”, each observable having two possible values, 0 =  “Yes” and 1  =  “No”. The analysis, formally, is precisely the same as above, except that one no longer uses the terms “hits” and “false alarms” (because “honesty” is not a signal objectively present in one of the two politicians and absent in another).

In quantum physics, a classical example falling within the same formal scheme as the examples above is one involving measuring the spin of a particle in a given direction. Let the experimenter choose one of two possible directions, 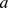 or 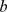 (unit vectors in space along which the experimenter sets a spin detector). If the particle is a spin-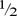 one, such as an electron, then the spin for each direction chosen can have one of two possible values, 1 =  “up” or 0 =  “down” (we need not discuss the physical meaning of these designations). These 1 and 0 are then the possible values of the observables 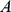 and 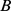 one associates with the two directions, and the analysis again consists in identifying the functions 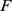, 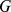, and 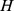.

## Theory

### 1 A brief account of conventional QT (with measurements of the first kind)

In QT, all entities operate in a *Hilbert space*, a vector space endowed with the operation of scalar product. The components of the vectors are *complex numbers*. We will assume that the Hilbert spaces to be considered are 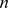-dimensional (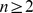), but the generalization of all our considerations to infinite-dimensional spaces is trivial. The scalar product of vectors 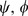 is 
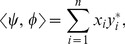
(6)where 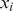 and 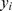 are components of 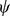 and 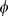, respectively, and the star indicates *complex conjugation*: if 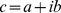, then 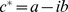. The length of a vector 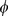 is defined as 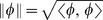.

Any observable 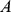 in this *n*-dimensional version of QT is represented by an 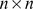 *Hermitian matrix* (or *operator*, the two terms being treated as synonymous in a finite-dimensional Hilbert space). This is a matrix with complex entries such that, for any 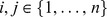, 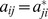. In particular, all diagonal entries of 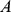 are real numbers. It is known from matrix algebra that any Hermitian matrix can be uniquely decomposed as 
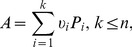
(7)where 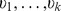 are pairwise distinct *eigenvalues* of 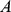 (all real numbers), and 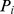 are *eigenprojectors* (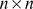 Hermitian matrices whose eigenvalues are zeros and ones). All eigenprojectors are *positive semidefinite*, i.e., for any nonzero vector 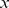, 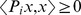, and they sum to the identity matrix, 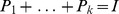. For any distinct 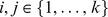, the eigenprojectors satisfy the conditions 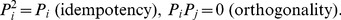
(8)


In QT, the distinct eigenvalues 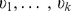 are postulated to form the set of all possible values 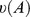. That is, as a result of measuring 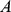 in any given trial one always observes one of the values 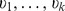. For simplicity (and because all our examples involve binary outcomes), in this paper we will only deal with the observables 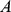 that have two possible values 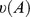, denoted 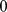 and 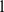. This means that all our observables can be presented as 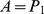, and 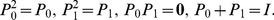
(9)


Each eigenvalue 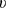 (0 or 1) has its *multiplicity* 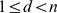. This is the dimensionality of the *eigenspace* 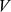 associated with 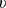, which is the space spanning the 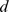 pairwise orthogonal *eigenvectors* associated with 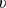 (i.e., the space of all linear combinations of these eigenvectors). Multiplication of 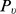 by any vector 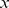 is the orthogonal projection of this vector into 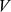. If 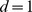, the eigenspace 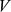 is the ray containing a unique unit-length eigenvector of 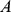 corresponding to 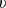. The eigenvalue 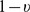 has the multiplicity 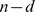, the dimensionality of the eigenspace 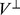 which is orthogonal to 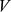. If both 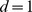 and 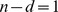 (i.e., 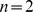), then 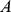 is said to have a *non-degenerate spectrum*. In this paper we assume the spectra are generally *degenerate* (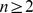).

The eigenvalues 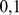 of 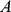 in a given trial generally cannot be predicted, but one can predict the probabilities of their occurrence. To compute these probabilities, QT uses the notion of a *state* of the system. In any given trial the state is unique, and it is represented by a unit length *state vector* 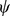. (For simplicity, we assume throughout the paper that the system is always in a *pure state*. This restriction is not critical for our analysis.) If the system is in a state 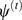 in trial 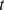, and the measurement is performed on the observable 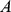, the probabilities of the outcomes of this measurement are given by 
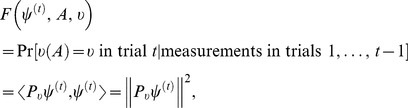
(10)where 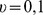. Note that these probabilities are conditioned on the previous observables, in trials 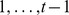, and their observed values.

Given that the observed outcome in trial 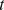 is 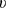, the state 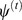 changes into 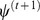 according to 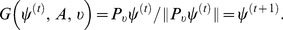
(11)


This equation represents the von Neumann-Lüders *projection postulate* of QT. The denominator is nonzero because it is the square root of 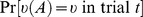, and (11) is predicated on 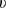 having been observed. The geometric meaning of 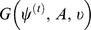 is that 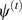 is orthogonally projected by 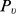 into the eigenspace 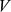 and then normalized to unit length.

Finally, the inter-trial dynamics of the state vector in QT (between 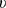 and the next observable, separated by interval 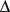) is represented by the *unitary evolution* formula 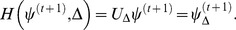
(12)


Here, 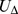 is a *unitary matrix*, defined by the property 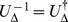, where, 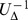 is the *matrix inverse* (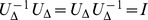), and 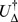 is the *conjugate transpose* of 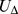, obtained by transposing 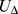 and replacing each entry 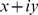 in it with its complex conjugate 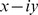. The unitary matrix 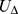 should also be made a function of inter-trial variations in the environment (such as variations in overall noise level, or other participants’ responses) if they are non-negligible. The identity matrix 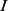 is a unitary matrix: if 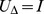, (12) describes *no inter-trial dynamics*, with the state remaining the same through the interval 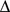. Note that the eigenvalue 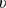 itself does not enter the computations. This justifies treating it as merely a label for the eigenprojectors and eigenspaces (so instead of 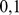 we could use any other labels).

*Remark* 1. In Pauli’s terminology [6], measurements described by (10)–(11)–(12) are called measurements of the first kind. The main distinguishing feature of such measurements is that two identical measurements “immediately following each other” (i.e., with 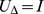) produce identical results. In Section 5 we consider a generalized formalism that include measurements of the first kind as a special case, but also covers a broad (arguably, most important) subclass of what Pauli calls measurements of the second kind (defined as all measurements not of the first kind, or not necessarily of the first kind).

### 2 Measurement sequences, evolution (in)effectiveness, and stability

In this section we introduce terminology and preliminary considerations needed in the subsequent analysis. Throughout the paper we will make use of the following way of describing measurements performed in successive trials: 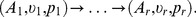
(13)


We call this a *measurement sequence*. Each triple in the sequence consists of an observable 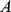 being measured, an outcome 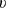 recorded (0 or 1), and its conditional probability 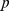. The probability is conditioned on the observables measured and the outcomes recorded in the previous trials of the same measurement sequence. Thus, 
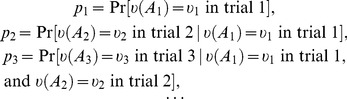
(14)


As we assume that the outcomes 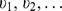 in a measurement sequence have been recorded, all probabilities 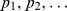 are positive if the measurement sequence exists. Recall that the observables 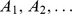 in a sequence are uniquely determined by the measurement procedures applied, 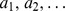, and that the outcomes (0 or 1) are eigenvalues of these observables.

Consider now the two-trial measurement sequence 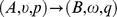, where 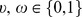. Let 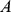 have the eigenprojectors 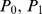, and 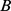 have the eigenprojectors 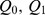. If the initial state of the system is 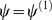, we have 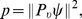 and 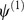 transforms into 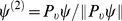. Assuming an interval 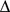 between the two trials, 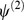 evolves into 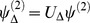. This is the state vector paired with 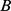 in the next measurement, yielding, with the help of some algebra, 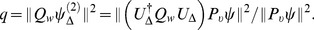
(15)


As a special case 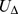 can be the identity matrix (no inter-trial changes in the state vector), and then we have 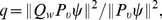
(16)because in this case 
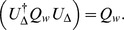
 It is possible, however, that the latter equality holds even if 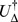 is not the identity matrix. In fact it is easy to see that this happens if and only if 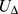 and 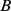 commute, i.e., 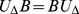. For the proof of this, see Lemma 1 in Text S2 Proofs. We will say that

#### Definition 1

A unitary operator 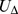 is *ineffective for an observable* 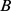 if the two operators commute, 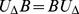.

The justification for this terminology should be transparent: due to Lemma 1, in the computation (15) of the probability 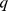 the evolution operator can be ignored, yielding (16). The notion of inefficiency of the evolution operator will play an important role in the analysis of repeated measurements below.

Our next consideration regards the set of all possible values of the initial state vector 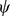 for a given measurement sequence. In the applications of QT in physics, this set is assumed to cover the entire Hilbert space in which they are defined. We are not justified to adopt this assumption in psychology, it would be too strong: one could argue that the initial states in a given experiment may be forbidden to attain values within certain areas of the Hilbert space. At the same time, it seems even less reasonable to allow for the possibility that the initial state for a given measurement sequence is always fixed at one particular value. The initial state vectors, as follows from both the QT principles and common sense, should depend on the system’s history prior to the given experiment, and this should create some variability from one replication of this experiment to another. This is important, because, given a set of observables, specially chosen initial state vectors may exhibit “atypical” behaviors, those that would disappear if the state vector were modified even slightly. It is known [7] that in physical systems very close states may have very different physical properties. We need therefore to confine our analysis to properties that, while they may not hold for the entire Hilbert space, are stable with respect to very small changes in the initial states for which they hold. This leads us to adopting the following

#### Stability Principle

*If* 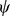 *is a possible initial state vector for a given measurement sequence in an n-dimensional Hilbert space, then there is an open ball* 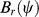 *centered at* 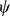 *with a sufficiently small radius r, such that any vector* 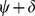 *in this ball, normalized by its length* 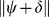 *, is also a possible initial state vector for this measurement sequence.*

#### Definition 2

A property of a measurement sequence is (or holds) *stable* for an initial vector 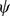, if it holds for all state vectors within a sufficiently small 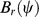.

Almost all our propositions below are proved under this stability clause, specifically by using the reasoning presented in Lemma 2 in Text S2 Proofs.

*Remark* 2. In Ref. [7] closeness is defined in terms of a measure called fidelity, which is different from the measure of closeness used in our stability principle. It is easy to show, however, that our measure topologically refines fidelity (i.e., any sequence of states converging to a given state in the sense of our measure also convergence to that state in the sense of fidelity).

### 3 Consequences for “a→a”-type measurement sequences

Using the definitions and the language just introduced, we will now focus on the consequences of (10)–(11)–(12) for repeated measurements with repeated responses, 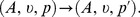
(17)


Consider an opinion-polling experiment, with questions like *a* =  “Is Bill Clinton trustworthy?” [5]. As argued for in Introduction, if the same question is posed twice, 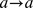, a typical respondent, who perhaps hesitated when choosing the response the first time she was asked 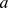, would now certainly be expected to repeat it, perhaps with some display of surprise at being asked the question she has just answered. This may not be true for all possible questions, but it is certainly true for a vast class thereof. Let us formulate this as

#### Property 1

*For some nonempty class of questions, if a question is repeated twice in successive trials (separated by one of a broad range of inter-trial intervals), the response to it will also be repeated.*

*Remark* 3. One may be tempted to dismiss this property as readily explained by the respondent’s “simply remembering” her previous answers. As argued in Conclusion, however, the availability of such common sense explanations is irrelevant for our analysis, as its purpose is to determine if the phenomena we consider can be explained in a unified mathematical language of QT.

If a question 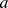 within the scope of Property 1 is represented by an observable 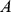, we are dealing with the measurement sequence (17) in which 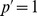. Such a measurement sequence does not disagree with the formulas (10)–(11)–(12). In fact it is even predicted by them if the intervening inter-trial evolution of the state vector is assumed to be ineffective. Indeed, (15) for the measurement sequence (17) acquires the form 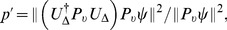
(18)and the inefficiency of 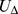 for 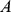 implies 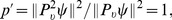
(19)because 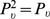 holds for all projection operators. We remind the reader that (10)–(11)–(12) define measurements of the first kind (see Remark 1). Our consideration is confined to these measurements until Section 5.

We see that ineffective evolution implies Property 1. As it turns out, under the stability principle, this implication can be reversed: effective inter-trial evolution is excluded for the observables representing the questions falling within the scope of Property 1. In other words, for all such questions, the unitary operators 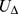 can be ignored in all probability computations. Let us say that

#### Definition 3

An observable 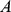 has the *Lüders property* with respect to a state vector 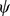 if the existence of the measurement 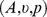 for this 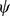 and an outcome 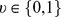 implies that the property 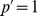 holds stable for this 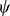 in the measurement sequence 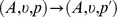.

In other words, the Lüders property means that an answer to a question (represented by 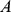) is repeated if the question is repeated, and that this is true not just for one initial state vector 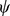, but for all state vectors sufficiently close to it.

*Remark* 4. Note that for the ineffective evolution (including the measurements that “immediately follow each other”) the Lüders property holds for all possible state vectors 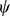. This was taken by Pauli [6] as the defining property of the measurements of the first kind. As argued at the introduction of the stability principle in Section 2, in psychology formulations involving “all possible initial states” would be unjustifiably strong.

We now can formulate our first proposition.

#### Proposition 1

*[repeated measurements] An observable* 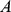 *has the Lüders property if and only if* 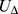 *in (12) is ineffective for* 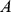.

See Text S2 Proofs for a formal proof. In the formulation of Property 1, the interval 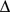 and the question represented by 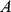 can vary within some broad limits, whence the inefficiency of 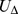 for 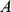 should also hold for each of these intervals combined with each of these questions.

We have to be careful not to overgeneralize the Lüders property and the ensuing inefficiency property. As we discussed in Introduction, one can think of situations where replications of a question may lead the respondent to “change her mind.” The most striking contrast, however, is provided by psychophysical applications of QT. Here, the inter-trial dynamics not only cannot be ignored, it must play a central role.

Let us illustrate this on an old but very thorough study by Atkinson, Carterette, and Kinchla [8]. In the experiments they report, each stimulus consisted of two side-by-side identical fields of luminance 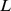, to one of which a small luminance increment 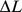 could be added, serving as the signal to be detected. There were three stimuli: 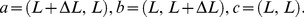
(20)


In each trial the observer indicated which of the two fields, right one or left one, contained the signal. There were thus two possible responses: Left and Right. An application of QT analysis to these experiments requires 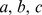 to be translated into observable 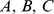, each with two eigenvalues, say, 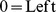 and 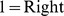. In the experiments we consider no feedback was given to the observers following a response. This is a desirable feature. It makes the sequence of trials we consider formally comparable to successive measurements of spins in quantum physics: measurements simply follow each other, with no interventions in between.

We are interested in measurement sequences 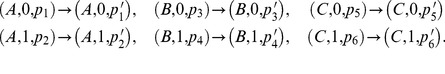
(21)


Recall that the probabilities 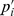 (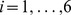) are conditioned on previous measurements, so that, e.g., 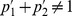 while 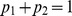. For each observer, the probabilities were estimated from the last 400 trials out of 800 (to ensure an “asymptotic” level of performance). The results of one of the experiments (with equiprobable 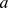 and 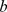), averaged over 24 observers, were as follows: 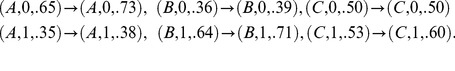
(22)


In accordance with Proposition 1, we should conclude that the inter-trial evolution (12) here intervenes always and significantly.

### 4 Consequences for “a→b→a”-type measurement sequences

Returning to the opinion polling experiments, consider the situation involving two questions, such as *a* =  “Is Bill Clinton honest?” and *b* =  “Is Al Gore honest?” The two questions are posed in one of the two orders, 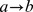 or 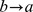, to a large group of people. The same as with asking the same question twice in a row, one would normally consider it unnecessary to extend these sequences by asking one of the two questions again, by repeating 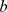 or 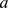 after having asked 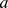 and 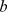. A typical respondent, again, will be expected to repeat her first response. We find it “almost certain” (the “almost” being inserted here because we cannot refer to any systematic experimental study of this obvious expectation) that from the nonempty (in reality, vast) class of questions falling within the scope of Property 1 one can always choose pairs of questions falling within the scope of the following extension of this property. (See Remark 3.)

#### Property 2

*Within a nonempty subclass of questions (and for the same set of inter-trial intervals) for which Property 1 holds, if a question a is asked following questions a and b (in either order), the response to it will necessarily be the same as that given to the question a the first time it was asked*.

As always, we replace 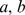 with observables 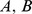, and use the following notation: the probability of obtaining a value 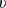 when measuring the observable 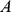 is denoted 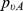, 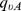, etc. (the letters 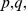 etc. distinguishing different measurements); we use analogous notation for the probability of obtaining a value 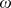 when measuring the observable *B*. Consider the measurement sequence 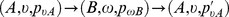
(23)


Property 2 implies that in these sequences 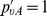 and 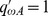. As it turns out, this property has an important consequence (assuming the two inter-trial intervals in the measurement sequences belong to the same class as 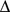 in Proposition 1).

#### Proposition 2

*[alternating measurements] Let A and B possess the Lüders property, and let the measurement sequences* 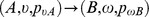 *exist for all* 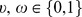 *, and some initial state vector* 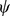 *. Then, in the measurement sequences (23), the property* 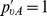 *holds stable for this* 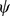 *if and only if A and B commute, AB*  =  *BA*.

In other words, if the probabilities 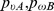 in 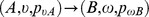 are nonzero for some 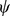, the sequences (23) exist with 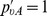 for all state vectors in a small neighborhood of 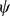 if and only if 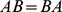. See Text S2 Proofs for a formal proof.

The commutativity of 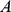 and 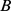 is important because it has an experimentally testable consequence.

#### Proposition 3

*[no order effect] If A and B possessing the Lüders property commute, then in the measurement sequences* 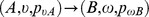 *and* 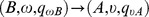 *, the joint probabilities of the two outcomes are the same, *

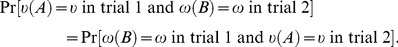
(24)


*Consequently*, 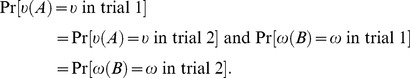
(25)


The proof of the proposition is given in Text S2 Proofs.

Equations (24)–(25) are empirically testable predictions. Moreover, if we assume that the questions like “Is Clinton honest” and “Is Gore honest” fall within the scope of Property 2 (and it would be amazing if they did not), these predictions are known to be de facto falsified.

#### Property 3

*Within a nonempty subclass of questions for which Property 2 holds (and for the same set of inter-trial intervals), the joint probability of two successive responses depends on the order in which the questions were posed.*

This “*question order effect*” has in fact been presented as one for whose understanding QT is especially useful: the empirical finding that (24) fails is explained in Ref. [9] by assuming that 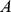 and 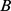 do not commute. In the survey reported by Moore [5], about 1,000 people were asked two questions, one half of them in one order, the other half in another. The results are presented for four pairs of questions, in the form 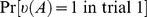 versus 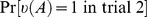, and analogously for *B*: 
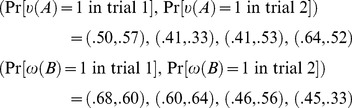
(26)


As we can see, for all question pairs, the probability estimates of Yes to the same question differ depending on whether the question was asked first or second. Given the sample size (about 500 respondents per question pair in a given order) the differences are not attributable to chance variation.

Properties 1, 2, and 3 turn out to be incompatible within the framework of the conventional QT (with measurements only of the first kind). We should conclude therefore that this formalism cannot be applied to the questions that have these properties without modifications.

### 5 Would POVMs work?

Are there more flexible versions (generalizations) of QT that could be used instead?

One widely used generalization of the conventional QT involves replacing the projection operators with *positive-operator-valued measures* (POVMs), see, e.g., Refs. [10], [11]. POVMs may but do not have to conform with (10)–(11)–(12). The generalized theory therefore involves measurements of both first and second kind.

The conceptual set-up here is as follows. We continue to deal with an *n*-dimensional Hilbert space (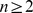). The notion of a state represented by a unit vector 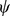 in this space remains unchanged. The generalization occurs in the notion of an observable. For experiments with binary outcomes, an observable 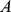 of the conventional QT is defined by 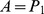, with eigenprojectors 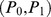 and eigenvalues 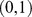. The eigenvalues themselves are not relevant insofar as they are distinct: replacing 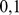 with another pair of distinct values amounts to trivial relabeling of the measurement outcomes. The information about the observable 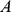 therefore is contained in the eigenprojectors 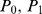. They are Hermitian positive semidefinite operators subject to the restrictions (9).

A generalized observable, or POVM, 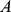 (continuing to consider only binary outcomes) is defined as a pair 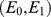 of Hermitian positive semidefinite operators in the *n*-dimensional Hilbert space, summing to the identity matrix *I*. In other words, the generalization from eigenprojectors 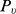 to POVM components 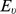 amounts to dropping the idempotency and orthogonality constraints, defined in (8).

Any component 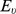 (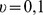) can be presented as 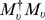, where 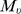 is some matrix and 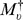 is its conjugate transpose. The representation 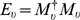 for a given 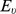 is not unique, but it is supposed to be fixed within a given experiment (i.e., for a given measurement procedure).

The measurement formulas specifying 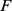 and 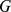 in (1)–(2) can now be formulated to resemble (10)–(11). The conditional probability of an outcome 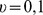 of the measurement of 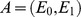 in state 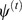 is 
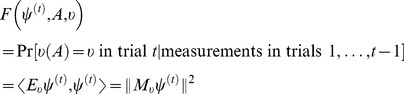
(27)


This measurement transforms 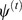 into 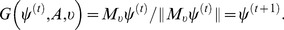
(28)


The formula for the evolution of the state vector between trials remains the same as for the conventional observables, (12).

It is easy to see that we no longer need to involve inter-trial changes in the state vector to explain the fact that, in psychophysics, a replication of stimulus does not lead to the replication of response. In a measurement sequence 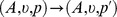, if 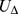 is the identity matrix, then 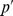 is given by 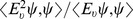. This value is generally different from 1, because 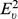, not necessarily an orthogonal projector, is generally different from 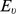.

This is interesting, as it suggests the possibility of treating psychophysical judgments and opinion polling within the same (evolution-free) framework. This encouraging possibility, however, cannot be realized: the theory of POVMs cannot help us in reconciling Properties 2 and 3 in opinion-polling, because POVMs with Lüders property cannot be anything but conventional observables. This is shown in the following.

#### Proposition 4

*[no generalization] A POVM* 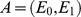 *has the Lüders property with respect to a state* 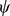 *if and only if* 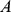 *is a conventional observable (i.e., it is a Hermitian operator, and its components* 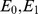 *are its eigenprojectors).*

See Text S2 Proofs for a formal proof.

Proposition 4 says that POVMs to be used to model opinion polling should be conventional observables, otherwise Property 1 will be necessarily contradicted. Put differently, the Lüders property effectively confines the measurements that can be considered within the framework of POVMs to those of the first kind. But then Propositions 1 and 2 are applicable, and they say that the inter-trial dynamics is ineffective, and that all the observables representing different questions within the scope of Property 2 pairwise commute. This, in turn, allows us to invoke Proposition 3, with the result that, contrary to Property 3, the order of the questions should have no effect on the response probabilities.

*Remark 5*. Not all measurements of the second kind can be described by POVMs (see, e.g., the discussion of quantum operations in Ch. 8 of Ref. [11]). One might argue that POVMs represent most “typical” quantum measurements. It remains to be seen, however, if other generalizations or modifications of QT would lead to different results (see Conclusion).

## Conclusions

Let us summarize. Both cognitive science and quantum physics deal with fundamentally probabilistic input-output relations, exhibiting a variety of sequential effects. Both deal with these relations and effects by using, in some form or another, the notion of an “internal state” of a system. In psychology, the maximally general version is provided by the probabilistic generalization of the old behaviorist S-O-R scheme: the probability of an output is a function of the input and the system’s current state (function 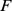 in (1)), and both the input and the output change the current state into a new state (function 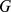 in (2)). If we discretize behavior into subsequent trials, then we need also a function describing how the state of the system changes between the trials (function 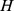 in (3)).

Quantum physics uses a special form of the functions 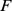, 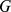, and 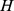, the ones derived from (or constituting, depending on the axiomatization) the principles of QT. Functions 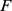 and 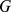 are given by (10)–(11) in the conventional QT, and by (27)–(28) in the QT with POVMs, with the inter-trial evolution in both cases described by (12). Nothing a priori precludes these special forms of 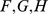 from being applicable in cognitive science, and such applications were successfully tried: by appropriately choosing observables and states, certain experimental data in human decision making were found to conform with QT predictions [3].

As this paper shows, however, QT encounters difficulties in accounting for some very basic empirical properties. In opinion polling (more generally, in all psychological tasks where stimuli/questions can be confidently identified by features other than those being judged), there is a class of questions such that a repeated question is answered in the same way as the first time it was asked. This agrees with the Lüders projection postulate, and renders the use of both the inter-trial dynamics of the state vector and the measurements of the second kind (at least those falling within the framework of the POVM theory) unnecessary: to have this property the questions asked have to be represented by conventional observables with ineffective inter-trial dynamics. In many situations, we also expect that for a certain class of questions the response to two replications of a given question remains the same even if we insert another question in between and have it answered. This property can only be handled by QT if the conventional observables representing different questions all pairwise commute, i.e., can be assigned the same set of eigenvectors. This, in turn, leads to a strong prediction: the joint probability of two responses to two successive questions does not depend on their order. This prediction is known to be violated for some pairs of questions. The explanation of the “question order effect” is in fact one of the most successful applications of QT in psychology [9], but it requires noncommuting observables, and these, as we have seen, cannot account for the repeated answers to repeated questions.

Our paper in no way dismisses the applications of QT in cognitive psychology, or diminishes their modeling value. It merely sounds a cautionary note: it seems that we lack a deeper theoretical foundation, a set of well-justified principles that would determine where QT can and where it must not be used. We should also point out that the problems identified in this paper are not unique to QT. For example, random utility theories also have difficulty explaining the trial to trial dependencies in answers to questions. If we assume that a response is based on a randomly sampled utility in each trial, then repeating the response will produce different random samples in each trial. That is why in the experiments designed to test random utility models questions never repeated back to back, and instead “filler trials” are inserted to make participants forget their earlier choice.

Clearly, the basic properties that we have shown to contravene QT can be “explained away” by invoking considerations formulated in traditional psychological terms. One can, e.g., dismiss the problem with repeated questions in opinion polling by pointing out that the respondents “merely” remember their previous answers and “simply” do not want to contradict themselves. One can similarly dismiss the question order effect by pointing out that the first question “simply” changes the context for pondering the second question, e.g., reminds something the respondent would not have thought of had the second question been asked first. These may very well be valid considerations. But if one allows for such extraneous to QT explanations, one needs to understand (A) why the same extraneous considerations do not intervene in situations where QT is successfully applicable, and (B) why one cannot stick to considerations of this kind and dispense with QT altogether.

A reasonable answer is that the value of QT applications is precisely in that it replaces the disparate conventional psychological notions with unified and mathematically rigorous ones. But then in those situations where we find QT not applicable one needs more than invoking these conventional psychological notions. One needs principles. Both in a psychophysical detection experiment and in opinion polling, participants may think of various things between trials, and previously presented stimuli/questions as well as previously given responses definitely change something in their mind, affecting their responses to subsequent stimuli/questions. Why then the applicability of QT is not the same in these two cases? Why, e.g., should the inter-trials dynamics of the state vector (or the use of POVMs in place of conventional observables) be critical in one case and ineffective (or unnecessary) in another?

One should also consider the possibility that rather than acting as switches distinguishing the situations in which (10)–(11) or (27)–(28) are and are not applicable, the set of the hypothetical principles in question may require a higher level of generality for the functions 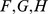. In Section 5 of Theory (see Remark 5) we mentioned the existence of measurements of the second kind falling outside the scope of POVMs. A serious and meticulous work is needed therefore to determine precisely what features of QT are critical for this or that (un)successful explanation. As an example, virtually any functions 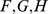 in the general formulas (1)–(2)–(3) predict the existence of the question order effect, and the functions can always be adjusted to account for any specific effect. The QQ constraint for the question order effect discovered by Wang and Busemeyer [9] means that, for any two questions 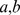 and any respective responses 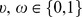, 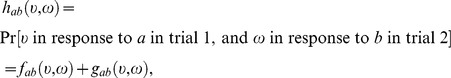
where 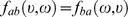 and 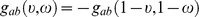. It follows then that 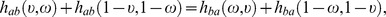
which is the QQ equation. Clearly, 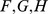 functions in (1)–(2)–(3) can be chosen so that 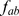 and 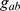 have the desired symmetry properties, and the QT version of 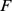 and 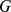 used in Ref. [9] (with ineffective 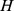) is only one way of achieving this. It is an open question whether one of many possible generalizations of this QT version may turn out more profitable for dealing with opinion polling.

## Supporting Information

Text S1 **Representative References.**
(PDF)

Text S2 **Proofs.**
(PDF)
