# Supplementary material for: Quantum Models for Psychological Measurements: An Unsolved Problem
Source: PLoS One. 2014 Oct 24;9(10):e110909. doi: 10.1371/journal.pone.0110909 (PMC4208824; doi:10.1371/journal.pone.0110909)
Supplement: Text S1 — Representative References. (PDF) [file pone.0110909.s001.pdf]

Text S1 Representative References

1. Acacio de Barros, J., Suppes, P. 2009 Quantum mechanics, interference, and the brain. *J. Math. Psych* **53**, 306-313.

2. Acacio de Barros, J. 2012 Joint probabilities and quantum cognition. In: *Quantum Theory: Reconsiderations of Foundations 6* (ed. A. Khrennikov, H. Atmanspacher, A. Migdall and S. Polyakov), *Special Section: Quantum-like decision making: from biology to behavioral economics, AIP Conf. Proc.* **1508**, pp. 98-104.

3. Accardi, L., Khrennikov, A., Ohya, M. 2008 The problem of quantum-like representation in economy, cognitive science, and genetics. In: *Quantum Bio-Informatics II: From Quantum Information to Bio-Informatics.* (ed. L. Accardi, W. Freudenberg, M. Ohya), p. 1-8. WSP, Singapore

4. Accardi, L., Khrennikov, A., Ohya, M. 2009 Quantum Markov model for data from Shafir-Tversky experiments in cognitive psychology. *Open Systems and Information Dynamics* **16**, 371-385.

5. Aerts, D., Aerts, S. 1994 Applications of quantum statistics in psychological studies of decision processes. *Foundations of Science*, **1**, 85-97.

6. Asano, M., Ohya, M. and Khrennikov, A. 2010 Quantum-like model for decision making process in two players game. *Foundations of Physics* **41**, 538-548.

7. Asano, M., Ohya, M., Tanaka, Y., Khrennikov, A. and Basieva, I. 2010 On application of Gorini-Kossakowski-Sudarshan-Lindblad equation in cognitive psychology. *Open Systems and Information Dynamics* **17**, 1-15.

8. Asano, M., Ohya, M., Tanaka, Y., Khrennikov, A. and Basieva, I. 2011 Dynamics of entropy in quantum-like model of decision making. *J. Theor. Biology* **281**, 56-64.

9. Atmanspacher H., Romer, H., Walach, H. 2002 Weak quantum theory: complementarity in physics and beyond. *Foundations of Physics* **32**, 379-406.

10. Atmanspacher, H., Filk, Th. and Römer, H. 2004 Quantum Zeno features of bistable perception. *Biological Cybernetics* **90**, 33-40.

11. Atmanspacher, H., Filk, Th. 2012 Temporal nonlocality in bistable perception. In: *Quantum Theory: Reconsiderations of Foundations - 6* (ed. A. Khrennikov, H. Atmanspacher, A. Migdall and

S. Polyakov), *Special Section: Quantum-like decision making: from biology to behavioral economics, AIP Conf. Proc.* **1508**, pp. 79-88.

12. Atmanspacher, H. and Filk, T. The Necker-Zeno model for bistable perception. *Topics in Cognitive Science*, in press.

13. Basieva, I., Khrennikov, A., Ohya, M. and Yamato, I. 2010 Quantum-like interference effect in gene expression glucose-lactose destructive interference. *Syst. and Synth. Biology*, 1-10.

14. Blutner R., Pothos E. M., Bruza P. 2013 A quantum probability perspective on borderline vagueness. *Topics in Cognition Science*, **5**, 711-736.

15. Busemeyer, J. R. and Bruza, P. D. 2012 *Quantum models of cognition and decision.* Cambridge Press.

16. Busemeyer, J. B., Wang, Z. and Townsend, J. T. 2006 Quantum dynamics of human decision making, *J. Math. Psychology* **50**, pp. 220-241.

17. Busemeyer, J. B. and Wang, Z. 2007 Quantum information processing explanation for interactions between inferences and decisions, In: *Quantum Interaction, AAAI Spring Symposium*, Technical Report SS-07-08 (ed. P.D. Bruza, W. Lawless, K. van Rijsbergen, D.A. Sofge), pp. 91-97. AAAI Press, Menlo Park, CA

18. Busemeyer, J. R., Matthews, M., and Wang, Z. 2006 A quantum information processing explanation of disjunction effects, In: *The 29th Annual Conference of the Cognitive Science Society and the 5th International Conference of Cognitive Science* (R. Sun and N. Myake), pp. 131-135. Mahwah, NJ. Erlbaum

19. Busemeyer, J. R., Santuy, E., Lambert-Mogiliansky, A. 2008 Comparison of Markov and quantum models of decision making, In: *Quantum interaction: Proceedings of the Second Quantum Interaction Symposium* (P. Bruza, W. Lawless, K. van Rijsbergen, D. A. Sofge, B. Coeke, S. Clark), pp. 68-74. London: College Publications

20. Busemeyer, J. R., Wang, Z., Lambert-Mogiliansky, A. 2009 Empirical comparison of Markov and quantum models of decision making. *Journal of Mathematical Psychology* **53 (5)**, 423-433.

21. Busemeyer, J. R., Pothos, E. M., Franco, R. and Trueblood, J. 2011 A quantum theoretical explanation for probability judgment errors. *Psychological Review* **118**, 193-218.

22. Busemeyer, J. R. and Bruza, P. D. 2012 *Quantum models of cognition and decision.* Cambridge Press.

23. Cheon, T. and Takahashi, T. 2010 Interference and inequality in quantum decision theory. *Phys. Lett. A* **375**, 100-104.

24. Cheon, T. and Tsutsui, I. 2006 Classical and quantum contents of solvable game theory on Hilbert space. *Phys. Lett. A* **348**, 147-152.

25. Conte, E., Todarello, O., Federici, A., Vitiello, F., Lopane, M., Khrennikov, A. and Zbilut, J. P. 2006 Some remarks on an experiment suggesting quantum-like behavior of cognitive entities and formulation of an abstract quantum mechanical formalism to describe cognitive entity and its dynamics. *Chaos, Solitons and Fractals* **31**, pp. 1076-1088.

26. Conte, E., Khrennikov, A., Todarello, O., Federici, A., Mendolicchio, L, Zbilut, J. P. 2008 A preliminary experimental verification on the possibility of Bell inequality violation in mental states. *Neuroquantology* **6**, pp. 214-221.

27. Conte, E., Khrennikov, A., Todarello, O., Federici, A., Mendolicchio, L, Zbilut, J. P. 2009 Mental state follow quantum mechanics during perception and cognition of ambiguous figures. *Open Systems and Information Dynamics* **16**, pp. 1-17.

28. Dzhafarov, E.N. & Kujala, J.V. 2012 Selectivity in probabilistic causality: Where psychology runs into quantum physics. *Journal of Mathematical Psychology* **56**, 54-63.

29. Dzhafarov, E.N. & Kujala, J.V. 2012 Quantum entanglement and the issue of selective influences in psychology: An overview. *Lecture Notes in Computer Science* **7620**, 184-195.

30. Dzhafarov, E.N., & Kujala, J.V. 2013 All-possible-couplings approach to measuring probabilistic context. *PLoS ONE* **8(5): e61712**. (DOI:10.1371/journal.pone.0061712).

31. Dzhafarov, E.N., & Kujala, J.V. 2014 On selective influences, marginal selectivity, and Bell/CHSH inequalities. *Topics in Cognitive Science* **6**, 121-128.

32. Fichtner, K.H., Fichtner, L., Freudenberg, W. and Ohya, M. 2008 On a quantum model of the recognition process. *QP-PQ: Quantum Prob. White Noise Analysis* **21**, 64-84.

33. Haven, E. and Khrennikov, A. 2009 Quantum mechanics and violation of the sure-thing principle: the use of probability interference and other concepts. *J. Math. Psychology* **53**, 378-388.

34. Haven, E. and Khrennikov, A. 2012 *Quantum Social Science*, Cambridge Press.

35. Haven, E. and Khrennikov, A. 2013 Quantum-like tunneling and levels of arbitrage. *International Journal of Theoretical Physics* **52**, 4083-4099.

36. Ishio, H., Haven, E. 2009 Information in asset pricing: a wave function approach. *Annalen der Physik* **18(1)**, 33-44.

37. Khrennikov, A. 2003 Quantum-like formalism for cognitive measurements. *Biosystems* **70**, 211-233.

38. Khrennikov, A. 2004 On quantum-like probabilistic structure of mental information. *Open Systems and Information Dynamics* **11 (3)**, 267-275.

39. Khrennikov, A. 2006 Quantum-like brain: Interference of minds. *BioSystems* **84**, 225–241.

40. Khrennikov, A. 2008 The quantum-like brain on the cognitive and subcognitive time scales. *J. Consciousness Studies* **15**, 39-77.

41. Khrennikov, A. 2009 Quantum-like model of cognitive decision making and information processing. *Biosystems* **95**, 179-187.

42. Khrennikov, A. 2010 *Ubiquitous quantum structure: from psychology to finance.* Springer, Heidelberg-Berlin-New York.

43. Khrennikova, P., Haven, E. and Khrennikov, A. 2013 An application of the theory of open quantum systems to model the dynamics of party governance in the US Political System. *International Journal of Theoretical Physics*, (DOI: DOI 10.1007/s10773-013-1931-6).

44. Lambert-Mogiliansky A., Busemeyer J. R. 2012 Quantum type indeterminacy in dynamic decision-making: Self-control through identity management. *Games* **3**, 97-118.

45. Ohya, M. and Volovich, I. 2011 *Mathematical foundations of quantum information and computation and its applications to nano- and bio-systems.* Springer, Heidelberg-Berlin-New.

46. Pothos, E. M., Busemeyer, J. R. 2009 A quantum probability explanation for violation of rational decision theory. *Proc. Royal. Soc. B* **276**, 2171-2178.

47. Pothos, E. M., Busemeyer, J. R. 2013 Can quantum probability provide a new direction for cognitive modeling? *Behavioral and Brain Sciences* **36**, 255-274.

48. Pothos, E. M., Busemeyer, J. R. and Trueblood, J. S. A quantum geometric model of similarity. *Psychological Review*, in press.

49. van Rijsbergen, K. 2004 *The geometry of information retrieval*. Cambridge: Cambridge University Press.

50. Yukalov S., Sornette D. 2011 Decision theory with prospect interference and entanglement. *Theory and Decision*, **70**, 283-328.

51. Wang, Zh., Busemeyer, J.R. 2013 A quantum question order model supported by empirical tests of an a priori and precise prediction. *Topics in Cognitive Sciences* **5**, 689-710.

52. Wang, Zh., Busemeyer, J.R., Atmanspacher, H. and Pothos, E. M. The potential of using quantum theory to build models of cognition. *Topics in Cognitive Science*, in press.
