# Supplementary material for: Quantum Models for Psychological Measurements: An Unsolved Problem
Source: PLoS One. 2014 Oct 24;9(10):e110909. doi: 10.1371/journal.pone.0110909 (PMC4208824; doi:10.1371/journal.pone.0110909)
Supplement: Text S2 — Proofs. (PDF) [file pone.0110909.s002.pdf]

Text S2 Proofs

Proposition 1 *(repeated measurements) An observable A has the Lüders property if and only if U_Δ in (12) is ineffective for A.*

Proof. The "if" part is demonstrated by (18)-(19). For the "only if" part, let the eigenprojector P correspond to the eigenvalue v of A. We have

$$p = \langle P\psi, \psi \rangle = \|P\psi\|^2 > 0,$$

and the next state vector is

$$\psi^{(2)} = \frac{P\psi}{\|P\psi\|}.$$

Following the evolution

$$\psi^{(2)} \to \psi_\Delta^{(2)} = U_\Delta \psi^{(2)},$$

the Lüders property implies

$$p' = \langle P\psi_\Delta^{(2)}, \psi_\Delta^{(2)} \rangle = 1,$$

or, equivalently,

$$\langle PU_\Delta \psi^{(2)}, U_\Delta \psi^{(2)} \rangle = \langle U_\Delta^\dagger P U_\Delta \psi^{(2)}, \psi^{(2)} \rangle = 1.$$

As $\|\psi^{(2)}\| = 1$, and $U_\Delta^\dagger P U_\Delta$ is an orthogonal projection, the lengths $\left\| U_\Delta^\dagger P U_\Delta \psi^{(2)} \right\|$ does not exceed 1. Therefore $\langle U_\Delta^\dagger P U_\Delta \psi^{(2)}, \psi^{(2)} \rangle = 1$ implies

$$U_\Delta^\dagger P U_\Delta \psi^{(2)} = \psi^{(2)},$$

or

$$U_\Delta^\dagger P U_\Delta P\psi = P\psi.$$

By the stability considerations (Lemma 2 below, with $X_1 = P$ to guarantee $p > 0$, and $Y = U_\Delta^\dagger P U_\Delta, Z = P$),

$$U_\Delta^\dagger P U_\Delta P = P.$$

Since P is Hermitian

$$P^\dagger = P \left(U_\Delta^\dagger P U_\Delta \right) = P = \left(U_\Delta^\dagger P U_\Delta \right) P,$$

so P and $U_\Delta^\dagger P U_\Delta$ commute. Now, $U_\Delta^\dagger (I-P) U_\Delta = I - U_\Delta^\dagger P U_\Delta$, and it commutes with $I-P$:

$$\left(I - U_\Delta^\dagger P U_\Delta\right)(I-P) = I - U_\Delta^\dagger P U_\Delta - P + \left(U_\Delta^\dagger P U_\Delta\right) P = I - U_\Delta^\dagger P U_\Delta - P + P\left(U_\Delta^\dagger P U_\Delta\right) = (I-P)\left(I - U_\Delta^\dagger P U_\Delta\right).$$

Let us choose an orthonormal basis $e_1, \ldots, e_n$ consisting of the eigenvectors of P, so that $e_1, \ldots, e_k$ are the eigenvectors associated with eigenvalue 1 (and then the rest of the e's are the eigenvectors of $I-P$ with eigenvalue 1). In this basis, P is a diagonal matrix with the first k diagonal entries 1, and the rest of them zero, and $I-P$ is a diagonal matrix with the last $n-k$ diagonal entries 1, and the rest of them zero. We have $Pe_i = e_i$, for $i \leq k$, and then

$$\left(U_\Delta^\dagger P U_\Delta\right) P e_i = \left(U_\Delta^\dagger P U_\Delta\right) e_i = e_i.$$

So, all $e_1, \ldots, e_k$ are eigenvectors of $U_\Delta^\dagger P U_\Delta$ with eigenvalues 1. Since $U_\Delta^\dagger P U_\Delta$ has the same eigenvectors $e_1, \ldots, e_n$ as P, it is a diagonal matrix with the first k diagonal entries 1. Analogously we find that $U_\Delta^\dagger (I-P) U_\Delta$ is a diagonal matrix with the last $n-k$ diagonal entries 1. Since these matrices add to I, the rest of the diagonal entries in $U_\Delta^\dagger P U_\Delta$ must be zero, and this means that $U_\Delta^\dagger P U_\Delta = P$. By Lemma 1, this means that U_Δ and A commute. □

Proposition 2 *(*alternating measurements*). Let A and B possess the Lüders property, and let the measurement sequences*

$$(A, v, p_{vA}) \to (B, w, p_{wB})$$

exist for all $v, w \in \{0, 1\}$, and some initial state vector ψ. Then, in the measurement sequences

$$(A, v, p_{vA}) \to (B, w, p_{wB}) \to (A, v, p'_{vA}),$$

the property $p'_{vA} = 1$ holds stable for this ψ if and only if A and B commute, $AB = BA$.

Proof. Let the eigenprojectors P and Q correspond to the eigenvalue 0 of A and B, respectively. Consider the measurement sequence

$$(A, 0, p_{0A}) \to (B, 0, p_{0B}) \to (A, 0, p'_{0A}).$$

Let the two inter-trial intervals be Δ_1 and Δ_2. Due to the Lüders property, each of the corresponding evolution operators U_1 and U_2 is ineffective for both A and B (commutes with any of their eigenprojectors).

Then the state vectors at the beginning of each trial are

$$\psi = \psi^{(1)} \to U_1 \frac{P_v \psi}{\|P_v \psi\|} = \psi^{(2)}_{\Delta_1} \to U_2 U_1 \frac{Q_w P_v \psi}{\|Q_w U_1 P_v \psi\|} = \psi^{(3)}_{\Delta_2}$$

and the corresponding probabilities are

$$p_{0A} = \|P\psi\|^2,\ p_{0B} = \frac{\langle QP\psi, P\psi\rangle}{\|P\psi\|^2},\ p'_{0A} = \frac{\langle PQP\psi, QP\psi\rangle}{\|QP\psi\|^2}.$$

The "if" part is proved by direct computation. If $AB = BA$, then $PQ = QP$, and we have

$$p'_{0A} = \frac{\langle PQP\psi, QP\psi\rangle}{\|QP\psi\|^2} = \frac{\langle QP\psi, QP\psi\rangle}{\|QP\psi\|^2} = 1.$$

We now prove the "only if" part. Denoting $\phi = QP\psi/\|QP\psi\|$, the condition

$$\langle P\phi, \phi\rangle = p'_{0A} = 1$$

implies $P\phi = \phi$, because $\|\phi\| = 1$, and $\|P\phi\|$ (the length of an orthogonal projection of ϕ) does not exceed 1. Hence

$$PQP\psi = QP\psi.$$

By the stability considerations (Lemma 2 below, with $X_1 = P, X_2 = PQP$ to guarantee $p_{0A} > 0, p_{0B} > 0$, and $Y = PQP, Z = QP$),

$$PQP = QP,$$

and, taking the conjugate transpositions,

$$PQP = (PQP)^\dagger = PQ.$$

So, $PQ = QP$. By simple algebra then, either of $P, I - P$ commutes with either of $Q, I - Q$, and this means that $AB = BA$. □

Proposition 3 (no order effect). *If A and B possessing the Lüders property commute, then in the measurement sequences*

$$(A, v, p_{vA}) \to (B, w, p_{wB}),$$

$$(B, w, q_{wB}) \to (A, v, q_{vA})$$

the joint probabilities of the two outcomes are the same,

$$p_{vA}p_{wB} = q_{wB}q_{vA}.$$

Consequently,

$$\Pr\left[v(A) = v \text{ in trial 1}\right] = \Pr\left[v(A) = v \text{ in trial 2}\right]$$

and

$$\Pr\left[w(B) = w \text{ in trial 1}\right] = \Pr\left[w(B) = w \text{ in trial 2}\right].$$

Proof. For the sequence $(A, v, p_{vA}) \to (B, w, p_{wB})$, we have

$$\psi = \psi^{(1)} \to U_A \frac{P\psi}{\|P\psi\|} = \psi^{(2)}_{\Delta_A}$$

and the probabilities are

$$p_{0A} = \|P\psi\|^2,$$

$$p_{0B} = \langle Q\psi^{(2)}_{\Delta_A}, \psi^{(2)}_{\Delta_A}\rangle = \langle QU_A \frac{P\psi}{\|P\psi\|}, U_A \frac{P\psi}{\|P\psi\|}\rangle = \frac{\langle QU_A P\psi, U_A P\psi\rangle}{\|P\psi\|^2}.$$

The joint probability is therefore

$$p_{0A}p_{0B} = \langle QU_A P\psi, U_A P\psi\rangle = \langle PQP\psi, \psi\rangle,$$

where we have used the commutativity of the unitary operators with the observables. Analogously, for the sequence $(B, w, q_{wB}) \to (A, v, q_{vA})$, we get

$$p_{0B}p_{0A} = \langle QPQ\psi, \psi\rangle.$$

But P and Q commute by Proposition 2, whence

$$PQP = QPQ.$$

This proves $p_{vA}p_{wB} = q_{wB}q_{vA}$. The other two equations follow by presenting the probabilities in them as sums of suitably chosen joint probabilities. □

Proposition 4 *(no generalization)A POVM $A = (E_0, E_1)$ has the Lüders property with respect to a state ψ if and only if A is a conventional observable (i.e., it is a Hermitian operator, and its components E_0, E_1 are its eigenprojectors).*

Proof. The "if" part is obvious: if A is a Hermitian operator, it has the Lüders property with respect to any state ψ.

We prove the "only if" part. Consider the measurement sequence

$$(A, v, p_v) \to (A, v, p'_v)\,,$$

with $\psi = \psi^{(1)}$. Since

$$p_v = \langle E_v\psi, \psi\rangle = \langle M_v\psi, M_v\psi\rangle = \|M_v\psi\|^2 > 0,$$

the next state vector (interjecting the unitary evolution operator U_Δ) is

$$\psi^{(2)}_\Delta = U_\Delta\psi^{(2)} = U_\Delta\frac{M_v\psi}{\|M_v\psi\|} = \frac{U_\Delta M_v\psi}{\|M_v\psi\|}.$$

Since $\|\psi^{(2)}_\Delta\| = 1$, it follows from Lemma 3 below that the equality $p'_v = \langle E_v\psi^{(2)}_\Delta, \psi^{(2)}_\Delta\rangle = 1$ implies $E_v\psi^{(2)}_\Delta = \psi^{(2)}_\Delta$, or

$$E_v U_\Delta M_v\psi = U_\Delta M_v\psi.$$

By the stability considerations (Lemma 2 below, with $X_1 = E_v$ to guarantee $p_v > 0$, and $Y = E_v U_\Delta M_v, Z = U_\Delta M_v$),

$$E_v\left(U_\Delta M_v\right) = \left(U_\Delta M_v\right).$$

Since

$$E_v = M_v^\dagger M_v = M_v^\dagger U_\Delta^\dagger U_\Delta M_v = \left(U_\Delta M_v\right)^\dagger\left(U_\Delta M_v\right),$$

we can apply Lemma 4 below (with $E_v = E$ and $U_\Delta M_v = S$) to establish that all eigenvalues of E_v are 0's and 1's. Therefore E_v is an orthogonal projector operator. A POVM (E_0, E_1) with both components orthogonal projectors is a conventional observable. □

Lemma 1. *For a unitary operator U and an observable A with eigenprojectors P_0, P_1, if U and A commute then $U^\dagger P_v U = P_v$ for $v = 0$ and $v = 1$; and if $U^\dagger P_v U = P_v$ for either $v = 0$ or $v = 1$, then U and A commute.*

Proof. U and A commute if and only if U commutes with P_1 (associated with eigenvalue 1), because $A = P_1$. We should prove therefore that

$$UP_1 = P_1 U \iff U^\dagger P_1 U = P_1 \iff U^\dagger P_0 U = P_0.$$

For the first equivalence, if $UP_1 = P_1 U$, then $U^\dagger P_1 U = U^\dagger U P_1 = P_1$; conversely, if $U^\dagger P_1 U = P_1$, then $UP_1 = UU^\dagger P_1 U = P_1 U$. For the second equivalence, if $U^\dagger P_v U = P_v$, then $U^\dagger P_{1-v} U = U^\dagger (I - P_v) U = I - U^\dagger P_v U = I - P_v = P_{1-v}$. □

Lemma 2. *Let $X_1, \ldots, X_n, Y, Z$ be some matrices. The statement*

$$\begin{gathered}\langle X_1 \psi, \psi \rangle > 0, \ldots, \langle X_n \psi, \psi \rangle > 0 \\ \Downarrow \\ Y\psi = Z\psi\end{gathered}$$

holds stable for ψ if and only if $Y = Z$.

Proof. The "if" part is trivial. For the "only if" part, by the definition of stability the initial state can be chosen as

$$\overline{\psi} = \frac{\psi + \delta}{\|\psi + \delta\|},$$

where $\delta \in B_r(0)$ (open ball of radius r centered at 0). By continuity considerations, r can be chosen sufficiently small for $\langle X_1 \psi, \psi \rangle, \ldots, \langle X_n \psi, \psi \rangle$ to remain positive. But $Y\overline{\psi} = Z\overline{\psi}$ any such $\overline{\psi}$, and we have

$$Y \frac{\psi + \delta}{\|\psi + \delta\|} = Z \frac{\psi + \delta}{\|\psi + \delta\|},$$

whence

$$Y\delta = Z\delta.$$

Since every vector is collinear to some δ, Y and Z coincide. □

Lemma 3. *Let (E_1, E_2) be a POVM. If $\langle E_v \phi, \phi \rangle = 1$ and $\|\phi\| = 1$, then $E_v \phi = \phi$.*

Proof. Writing $E_v\phi = c\phi + \gamma$, with $\gamma \perp \phi$, we see that $\langle E_v\phi, \phi\rangle = 1$ implies $c = 1$. Choose an orthonormal basis $(e_1, \ldots, e_n)$ in the Hilbert space so that $\phi = e_1$. In this basis $\gamma = \sum_{i=2}^{n} u_i e_i$. Assume that $\gamma \neq 0$, and let, with no loss of generality, $u_2 \neq 0$. The components E_v in this basis is

$$E_v = \begin{pmatrix} 1 & \overline{u_2} & \ldots & \overline{u_n} \\ u_2 & \ldots & \ldots & \ldots \\ \vdots & \vdots & \ddots & \vdots \\ u_n & \ldots & \ldots & \ldots \end{pmatrix},$$

because when multiplied by $\phi = e_1 = (1, 0, \ldots, 0)^\top$, it should yield $e_1 + \sum_{i=2}^{n} u_i e_i = \phi + \gamma$. Then the other component in this basis is

$$E_{1-v} = \begin{pmatrix} 0 & -\overline{u_2} & \ldots & -\overline{u_n} \\ -u_2 & \ldots & \ldots & \ldots \\ \vdots & \vdots & \ddots & \vdots \\ -u_n' & \ldots & \ldots & \ldots \end{pmatrix},$$

because $E_v + E_{1-v} = I$. But with $u_2 \neq 0$, the leading principal minor

$$\begin{vmatrix} 0 & -\overline{u_2} \\ -u_2 & \ldots \end{vmatrix} < 0,$$

which contradicts the requirement that E_{1-v} be positive semidefinite. This contradiction shows that $\gamma = 0$. □

Lemma 4. *Let $E = S^\dagger S$ be a component of a POVM, and let $ES = S$. Then all eigenvalues of E are 0's and 1's.*

Proof. Since E is Hermitian, we can select a basis consisting of its eigenvectors. In this basis matrix E is diagonal with the diagonal elements $\lambda_1, ..., \lambda_n$ (the eigenvalues of E). Suppose that one of these elements, say λ_1, is not 1. From $ES = S$ it follows that $\lambda_1 s_1 = s_1$, where s_1 is the first row of S. Therefore s_1 of S consists of zeros. But since $E = S^\dagger S$, we have $\lambda_1 = \langle s_1, s_1\rangle = 0$. This proves that $\lambda_1, ..., \lambda_n$ consists of 0's and 1's. □
